# How does spaceflight affect the acquired immune system?

**DOI:** 10.1038/s41526-020-0104-1

**Published:** 2020-05-07

**Authors:** Taishin Akiyama, Kenta Horie, Eiichi Hinoi, Manami Hiraiwa, Akihisa Kato, Yoichi Maekawa, Akihisa Takahashi, Satoshi Furukawa

**Affiliations:** 1 Laboratory for Immune Homeostasis, RIKEN Center for Integrative Medical Sciences, Yokohama, 230-0045 Japan; 2 Laboratory of Pharmacology, Department of Bioactive Molecules, Gifu Pharmaceutical University, Gifu, Japan; 3 United Graduate School of Drug Discovery and Medical Information Sciences, Gifu University, Gifu, Japan; 4 Laboratory of Molecular Pharmacology, Division of Pharmaceutical Sciences, Kanazawa University Graduate School, Kanazawa, Ishikawa Japan; 5 Division of Molecular Virology, Department of Microbiology and Immunology, The Institute of Medical Science, The University of Tokyo, Minato-ku, Tokyo, 108-8639 Japan; 6 The Institute of Medical Science, The University of Tokyo, Minato-ku, Tokyo, 108-8639 Japan; 7 Research Center for Asian Infectious Diseases, The Institute of Medical Science, The University of Tokyo, Minato-ku, Tokyo, 108-8639 Japan; 8 Department of Parasitology and Infectious Diseases, Gifu University Graduate School of Medicine, 1-1 Yanagido, Gifu, 501-1194 Japan; 9 Domain of Integrated Life Systems, Center for Highly Advanced Integration of Nano and Life Sciences (G-CHAIN), Gifu University, 1-1 Yanagido, Gifu, 501-1194 Japan; 10 Gunma University Heavy Ion Medical Center, Maebashi, Gunma 371-8511 Japan; 11 Japan Aerospace Exploration Agency (JAXA), Tsukuba, Ibaraki 305-8505 Japan

**Keywords:** Adaptive immunity, Health care

## Abstract

The impact of spaceflight on the immune system has been investigated extensively during spaceflight missions and in model experiments conducted on Earth. Data suggest that the spaceflight environment may affect the development of acquired immunity, and immune responses. Herein we summarize and discuss the influence of the spaceflight environment on acquired immunity. Bone marrow and the thymus, two major primary lymphoid organs, are evidently affected by gravitational change during spaceflight. Changes in the microenvironments of these organs impair lymphopoiesis, and thereby may indirectly impinge on acquired immunity. Acquired immune responses may also be disturbed by gravitational fluctuation, stressors, and space radiation both directly and in a stress hormone-dependent manner. These changes may affect acquired immune responses to pathogens, allergens, and tumors.

Astronauts experience hostile environmental changes during spaceflight, including microgravity, high doses of radiation, psychological stress induced by constraints and fears, and hypergravity during launching and landing. These factors reportedly affect many physiological systems in the body^1–4^. Several studies suggest that the immune system may be disturbed by spaceflight^2–4^. The immune system as a whole encompasses two main components, innate immunity and acquired immunity. Acquired immune responses are critical for inflammation, immunity against infection, and tumor immunity. In this review, we discuss how spaceflight environments may influence the development and functions of acquired immunity.

## Acquired immune system and its development

Lymphocytes play central roles in acquired immune responses. Almost all lymphocytes, including T cells and B cells are originally derived from hematopoietic stem cells in the bone marrow^5^. While B cells mature in the bone marrow, the progenitors of T cells from bone marrow mature into T cells in the thymus. Mature T and B cells migrate from primary lymphoid organs and are distributed to the various peripheral organs of the body^6^. Activation of the innate immune system by pathogen invasion triggers T-cell activation and differentiation via antigen presentation and the activities of various cytokines^7^. Activated effector T cells further activate B cells and macrophages during immune responses, and can also directly kill virus-infected cells^8,9^. After pathogens are eliminated, proportions of antigen-specific T cells and B cells are converted into immunological memory cells that contribute to effective and long-lasting immunity against those pathogens^10^. Spaceflight may affect the initial development of lymphocytes, as well as their antigen-specific responses and the development of lymphocytic immunological memory, and thereby disrupt acquired immune responses^11,12^.

## Impact of spaceflight on the development of acquired immunity

Lymphoid organs are classified into primary lymphoid organs and secondary lymphoid organs, and both are required for the development and maintenance of efficient immune responses. Because the impact of spaceflight on lymphoid organ homeostasis is difficult to evaluate in humans, many spaceflight and ground-based model experiments have been conducted using rodents^13–19^.

The differentiation and maturation of B cells, myeloid cells, erythrocytes, hematopoietic stem cells (HSCs), and other progenitor cells occurs in the bone marrow, and bone is influenced by reduced gravity during spaceflight^20–22^. Therefore, spaceflight may change the bone marrow environment, thereby affecting immune systems. The bone marrow environment is composed of cells of hematopoietic origin (i.e., osteoclasts and macrophages), and non-hematopoietic stroma cells such as osteoblasts, fibroblasts, endothelial cells, and adipocytes^23^.

**Fig. 1:**
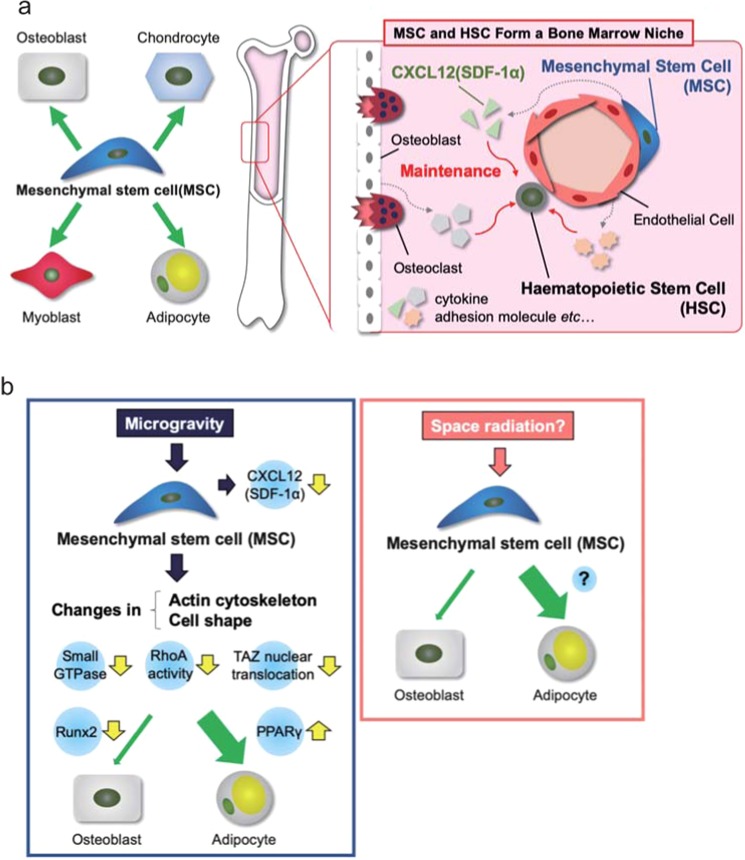
Functions and differentiation of mesenchymal stem cells (MSCs) in bone marrow. : **a** Function and differentiation of MSCs in the homeostatic condition. **b** Alteration in differentiation of MSCs by microgravity and space radiation.

Mesenchymal stem cells (MSCs) are a source of osteoblasts, chondrocytes, and adipocytes in the bone tissue^24^ (Fig. 1a). They also regulate the functions of HSCs and other hematopoietic cells as niche cells^23^. Astronauts and patients who are bedridden long-term with little to no gravitational stimulation of their bones exhibit a sharp decrease in bone mass and immune function. This can be prevented via mechanical loading, such as that imparted during exercise^25^. The role played by MSCs in dysfunction caused by microgravity environments is largely unclear, but associations between MSC characteristics and environmental gravitational changes have been reported (Fig. 1b)^26,27^. Microgravity inhibits osteogenic differentiation and increases the adipogenic differentiation of MSCs, at least partly via the inhibition of Runx2 expression and the promotion of PPARγ expression^26^. An imbalance in MSC lineage commitment to osteogenesis or adipogenesis is caused by changes in the actin cytoskeleton and cell shape via small GTPase, RhoA, and the transcriptional coactivator TAZ under microgravity conditions^26,27^.

MSCs and HSCs form a unique niche in the bone marrow microenvironment that regulates the stemness and function of stem cells and their lineage commitment by producing favorable factors^23^. In human MSCs microgravity inhibits the expression of the chemokine CXCL12 (also known as SDF-1α), which is essential for maintaining HSC stemness and function as well as their retention in bone marrow, their quiescence, and their repopulating capacity^28^. This suggests that MSC dysfunction may be involved in the apparent decline in immune function and/or “space anemia” reported in astronauts via interaction with HSCs in the bone marrow microenvironment.

Regenerative medicine using MSCs has recently received significant attention. Fine tuning of the duration of exposure to simulated microgravity in addition to differentiation-inducing conditions can control MSC-lineage commitment to multiple functional cell types including osteoblasts (osteogenesis), chondrocytes (chondrogenesis), adipocytes (adipogenesis), endothelial cells (angiogenesis), and neurons (neurogenesis)^29–31^. Moreover, human MSCs cultured under simulated microgravity retain stem cell characteristics and the ability to differentiate into hyaline cartilage following transplantation^32^. A previous study in mice suggests that space radiation, which is a major environmental stressor in humans in space, may affect MSC differentiation into adipocytes^33^. In that study, MSCs in irradiated mice preferentially differentiated into adipocytes in the bone marrow. Elucidation of the mechanisms underlying the biological responses of MSCs to environmental stressors in humans in space may contribute to advances in regenerative medicine using MSCs, as well assisting the design of strategies to prevent bone loss in astronauts and patients with age-related and disuse-related osteoporosis.

A bone marrow environment is required for B-cell generation. Although B-cell frequency was reportedly not changed immediately after spaceflight, it was significantly reduced 1 week after landing^34^. Spaceflight did not affect the immunoglobulin repertoires of mice after short-time spaceflight^35^. Consistently, in a recent study B-cell homeostasis was maintained in astronauts during long-term spaceflight^36^. These data suggest that the influence of spaceflight on B-cell development and function may be limited.

The thymus is a primary lymphoid organ and generates almost all of the T cells in the body^37^, and various physiological and psychological stressors can cause it to undergo atrophy^38–40^.

**Fig. 2:**
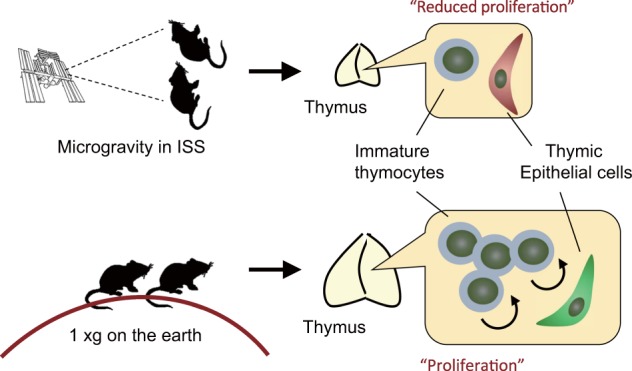
Influence of spaceflight on the murine thymus. : In mice, spaceflight causes thymic atrophy that is most likely due to reduced cell proliferation.

It is therefore not surprising that hostile environmental changes associated with spaceflight cause thymic atrophy (Fig. 2), and thereby may affect thymic functions. Impairment of thymic functions by spaceflight was suggested in a study that investigated human blood samples^41^. The recombination of T-cell antigen receptor genes during T-cell development in the thymus generates episomal circles containing the excised DNA (T-cell receptor excision circles; TRECs). As a result, naive T cells from the thymus can be detected via PCR analysis of TRECs^42^. TREC PCR analysis of blood samples from astronauts reportedly indicated that T-cell output from the thymus is reduced after spaceflight^41^, suggesting impairment of T-cell development in the thymus.

In addition to human sample analysis, the influence of spaceflight on the thymus has been investigated in rodents. Early studies performed during missions of ~2 weeks in space revealed that spaceflight caused an involution of the murine thymus^16,43^. In another relatively long-term spaceflight, there was also a reduction in murine thymic mass^18^. These data strongly suggest that spaceflight induces a thymic atrophy. Because mice would experience various kinds of environmental changes during spaceflight, however, it is difficult to determine what factors contribute to spaceflight-induced thymic atrophy.

The Japan Aerospace Exploration Agency (JAXA) recently developed an experimental platform in which mouse cages can be centrifuged to control the gravity experienced in the International Space Station (ISS)^22,44^. The thymi of mice exposed to 1 g in the ISS were recently analyzed^45^. Interestingly, exposure to experimentally imposed 1 g significantly alleviated spaceflight-induced thymic atrophy. Thus, altered gravity is presumably involved in thymic atrophy during spaceflight. Moreover, that result suggests that 1 g exposure may be an effective countermeasure against spaceflight-induced thymic atrophy. RNA sequencing analysis revealed down-regulation of genes regulating the G2/M phase of the cell cycle in murine thymi after spaceflight, suggesting that impairment of thymic cell proliferation may induce thymic involution in mice during spaceflight (Fig. 2).

In addition to a reduction in cell cycle regulating genes, the thymic microenvironment is reportedly affected by spaceflight^45^. During T-cell differentiation in the thymus interactions between T cells and thymic epithelial cells (TECs) are critical^46^. TECs are largely divided into cortical TECs and medullary TECs (mTECs). TECs expressing keratin-5 (Krt5; Krt5 TECs) are normally localized in the medullar region of the thymus, and are therefore classified as mTECs. Interestingly, spaceflight caused mislocalization of Krt5 TECs in the cortex region, implying that spaceflight affected properties of TECs. Given that TECs control the development and selection of thymocytes, it is possible that the disruptive effects of spaceflight on TECs indirectly caused a reduction in thymocytes. These changes in thymic gene expression and TEC localization associated with spaceflight are also alleviated by exposure to 1 g in the ISS, suggesting a critical role of altered gravity on these phenomena.

## Impact of spaceflight environment on immune cell responses

In addition to effects on immune development, spaceflight appears to lead to various other changes in immune responses. Samples from astronauts have been used to investigate the effects of spaceflight on the immune system^12,47–55^. In a seminal study in the early 1980s it was reported that neutrophils were elevated and eosinophils were reduced in the peripheral blood of astronauts who underwent spaceflight in the space shuttle^56^. Moreover, in vitro activation of T-cells prepared from astronauts was significantly reduced^57,58^. Further studies revealed that spaceflight affects various immune parameters such as the distribution of leukocytes^12,47^, granulocyte and monocyte function^47,52^, natural killer cell function^53^, and cytokine levels in plasma^49,54^ and in response to stimuli^12,55^.

Given that many kinds of environmental changes and stressors are induced during spaceflight, each factor may directly disturb immune cell numbers and functions via individual mechanisms. In addition to direct effects, spaceflight-associated stressors increase levels of stress hormones (*i.e*., cortisol, dehydroepiandrosterone, epinephrine, and norepinephrine) via activation of hypothalamic-pituitary-adrenal and sympathetic-adrenal-medullary axes^59^. Because these stress hormones are known to affect immune cells^59^, modulation of the immune system by spaceflight may be partly due to these humoral factors. Because of this complexity, the precise mechanisms underlying the effects of spaceflight remain to be determined.

Gravity change is one of the typical events that occurs during spaceflight, and it may influence immune systems by both direct mechanisms and indirect mechanisms via the induction of stress hormones. Spaceflight has high associated operating costs, therefore in addition to spaceflight experiments several ground-based models of spaceflight have been developed to investigate the effects of microgravity. Experimental methods incorporating low-gravity environments such as parabolic flight, a simulated microgravity environment such as a clinostat, long-term bed rest, and hindlimb suspension in experimental animals have been employed. It is necessary to carefully consider the extent to which these experimental methods actually reflect microgravity.

Tauber et al.^60^ reported that cell surface expression of CD3 and IL-2 receptor on non-activated T cells in human peripheral blood decreased just 20 s after low-gravity induction in a parabolic flight study. In addition, activated T cells placed in a two-dimensional clinostat as a simulated microgravity environment for 5 min exhibited reduced surface expression of CD3, reduced ZAP-70, and increased histone H3 acetylation. When these changes are induced in T cells in an actual microgravity environment, TCR signaling may be attenuated or modulated, markedly affecting immune responses. Importantly, because the influence of steroid hormones can be excluded in this system, the result indicated that the immune cell itself responds to gravitational fluctuation, suggesting the existence of a gravity sensor in immune cells.

Murine hindlimb suspension is a ground-based model primarily used to study the effects of microgravity on muscles and bones^17^. Stress during hindlimb unloading seems to be similar to spaceflight-induced stress with regard to causing activation of the sympathetic nervous system and the hypothalamic-pituitary-adrenal axis, thereby disrupting systemic immune responses^17^. Both CD4 T-cell and CD8 T-cell responses to mitogenic stimulation were reduced in mice that had undergone hindlimb suspension^61^. Notably however, hindlimb suspension caused a reduction in CD4 T cells and an increase in CD8 T cells in the spleen^61^. The reason for this apparent inconsistency remains unknown, but one possibility is that CD4 T cells may be more sensitive to stress induced by hindlimb suspension than CD8 T cells, and the increase in CD8 T cells may be due to homeostatic proliferation that is induced by reduction in the overall T-cell population. Notably, given that hindlimb suspension is not a perfect model for studying systemic in vivo effects, further research incorporating spaceflight experiments will be necessary to facilitate definitive conclusions.

## Effects of spaceflight on immunity and the onset of disease

There have been no reports of astronauts developing serious infectious diseases during spaceflight or after returning, but it is currently unclear whether long-term exposure to continuous microgravity can promote infectious diseases. Because astronauts who are selected for space travel are both mentally and physically healthy, it is highly probable that the modulation of the immune system due to gravitational fluctuations may be affected by their robustness or homeostatic mechanisms. Alternatively, an immune system that has been modulated by a period of stay may still have a sufficient margin until the onset of serious infection. Notably, however, in a previous study astronauts who stayed in a space shuttle for a short period (12–16 days) before, during, and after the flight exhibited reactivation of latent viral infections^49^.

Approximately 70–95% of the global population is estimated to be infected with at least one of the nine known human herpes viruses (HHVs)^62^. The vast majority of HHV infections are considered clinically asymptomatic phases of infection referred to as latency^62^. However, space is expected to be a high-risk environment with regard to disturbing the symbiotic relationship between humans and latent infectious viruses. Reactivation and/or shedding of latent HHVs such as Varicella-zoster virus (VZV), EBV, and human cytomegalovirus has been reported in astronauts during the Russian Soyuz and ISS missions^49,59,63^. Furthermore, the National Aeronautics and Space Administration (NASA) reported that (i) VZV was detected in the saliva of ~50% of astronauts on flight and approximately 60% of astronauts during the flight phase of the ISS; and (ii) astronauts occasionally experienced cold sores, which are usually caused by herpes simplex virus type-1 (HSV-1), although the occurrence of HSV-1 in saliva was rare (https://humanresearchroadmap.nasa.gov/Evidence/reports/Host_Micro.pdf).

**Fig. 3:**
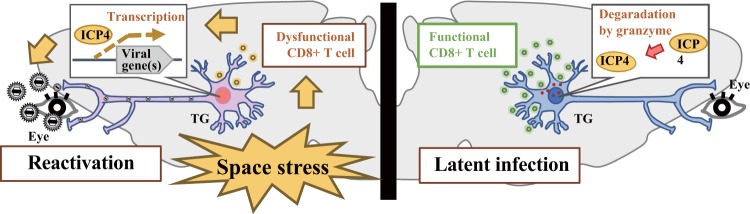
Reactivation of latent virus during spaceflight. Space stressors may cause dysfunction of CD8 T cells that in turns induces reactivation of HSV-1.

HSV-1 and VZV are neurotropic human alpha herpes viruses. After epithelial entry viral particles enter a plexus of sensory neurons, gaining access to neuronal axons. Once these viruses reach the neuronal body, effector memory CD8 and CD4 lymphocytes establish residence, and the viral genomes persist in latency in the trigeminal ganglion or dorsal root ganglion^64,65^. The immune response plays an important role in the ganglia during infection with HSV-1^66^. HSV-1 latency in sensory ganglia is characterized by persistent CD8 T-cell infiltration^67^, and the recovery of infectious viral particles from ex vivo explants of latently infected murine trigeminal ganglions following ocular infection is completely inhibited in the presence of CD8 T cells^68^. Furthermore, depletion of CD8 T cells from latently infected neuronal nodes can promote HSV-1 reactivation^68^. Notably, Knickelbein et al.^69^ reported that granzyme B release by CD8 T cells inhibited HSV-1 reactivation by degrading the essential viral transcription factor HSV-1 infected-cell polypeptide 4 (Fig. 3). From these findings, it is reasonable to conclude that space-induced impairment of adaptive immunity may be implicated in establishing HSV-1 and/or VZV latency.

Vaccines are an effective strategy for preventing viral diseases. No approved HSV-1 vaccine exists but a live attenuated VZV derived from Oka^70^ and a subunit vaccine containing VZV glycoprotein E and the AS01B adjuvant system^71^ are available to prevent both chickenpox and herpes zoster. The development of vaccines for other HHVs is desirable for administration to humans living in space. In the future, space travelers may utilize space-specific vaccines. Although the individual effects of the extreme stressors experienced in space such as gravity changes or radiation on the symbiotic relationship between humans and HHVs have not been elucidated, changes in the regulation of the host immune system are considered an important factor.

Because experimental infection in a spaceship would be problematic, some model ground-based experiments have been performed to investigate the influence of spaceflight-type environments on infectious immunity. Weisman et al.^72^ reported a synergistic effect of hindlimb suspension and radiation on the control of bacterial infection. The combination of hindlimb suspension and proton irradiation significantly increased the lethality of bacterial infection in mice after intraperitoneal inoculation with *Pseudomonas aeruginosa* above that of either treatment alone. A similar phenomenon was observed with the combination of hindlimb suspension and gamma irradiation. In the combined group there was almost no increase in granulocytes in blood against the infection. Total serum corticosterone was highest in the combined group. Zhou et al.^71^ reported similar effects of a different combined treatment (complex stress) on the control of intestinal microbiota. Serum lipopolysaccharides (LPS) increased 6 h after proton beam irradiation or hindlimb suspension. Because LPS elevations in serum may be derived from Gram-negative bacteria in the intestinal tract, these treatments resulted in a breakdown in the integrity of the intestinal epithelium. Moreover, the combined treatment of hindlimb suspension and irradiation exerted synergistic effects on LPS elevation. LPS elevation was transient after a single treatment, but it continued for at least 4 days after irradiation in the combined treatment group. In conjunction with the increase in serum LPS concentrations, serum LPS binding protein and soluble CD14 also increased. Furthermore, the inflammatory cytokines IL-6, tumor necrosis factor-α, and IFN-α were elevated. So-called LPS tolerance is induced by continuous stimulation with LPS. Spleen cells from mice that underwent 13 days of spaceflight reportedly exhibited reduced tumor necrosis factor-α production and low blastogenesis in response to LPS stimulation^15^. LPS tolerance may occur due to exposure to LPS, but reported spleen cell responses to mitogenic stimuli such as LPS vary between studies, thus further investigation is required in this regard.

In addition to compromised immune responses, spaceflight may affect the onset and progression of immunological conditions such as allergy and autoimmunity. Some astronauts experience allergy-like symptoms during spaceflight^73^. Moreover, skin rashes and hypersensitivity frequently occur in astronauts^74^. These reports suggest dysregulation of immune and inflammatory responses by spaceflight environments. The mechanisms underlying this dysregulation remain unclear. Various stressors can be risk factors for such symptoms^75^, thus it is possible that psychological stress during spaceflight indirectly contributes to their onset and/or progression. To date there is no clear evidence that spaceflight is associated risk of developing autoimmunity.

## Space-induced impairment of immunity: will it increase the risk of cancer?

The acquired immune system plays a major role in preventing tumor growth and metastasis, suggesting that immune system aberrations caused by stressors associated with space travel should be considered when estimating the risk of cancer-related mortality. Interestingly, studies to date have not detected significant differences in cancer mortality between astronauts and non-astronauts^76–79^ or between deep spaceflight astronauts and non-flight astronauts^80^. For reasons that have not been elucidated, astronauts do reportedly exhibit significantly higher rates of accidental death than non-astronauts^76–80^. Notably, it may be too early to draw definitive conclusions on health risks in astronauts, due to the small numbers surveyed and the fact that they tend to spend relatively short periods of time in space. Similarly, due to a lack of relevant information we cannot predict how travelling to the Moon or Mars, or long periods of time in deep space may affect health, including the risk of cancer.

Over the past 20 years risk assessments associated with space radiation have mainly focused on cancer. Space radiation can certainly damage DNA and induce genetic changes that may lead to cancer^81,82^. For example, damage such as double-strand break tracks have been detected in the nuclei of cells exposed to space radiation^82^, and increased frequencies of chromosomal aberrations have been detected in lymphocytes from astronauts during and after spaceflight. Many cancers may not emerge until years after the initial contributory genetic insults, which is relevant to a Mars mission with a potential duration of approximately 3 years because space-induced cancer may be detected after, but not during, spaceflight. Of course, astronauts can receive standard cancer treatment after spaceflight.

In addition to cancer risk assessment, NASA has recently increased its focus on space-induced central nervous system and cardiovascular diseases. The galactic cosmic ray simulator developed by NASA^83^ enables rapid switching between various high-energy ion sources, more closely simulating the radiation exposure experienced in space. The NASA space radiation program has encouraged researchers to investigate exposure to simulated solar particle events and galactic cosmic rays as part of their space radiation research. In the past, space-induced cancer risk assessment has been based on radiation quality and quantity, but the added risk of exposure to space radiation under microgravity conditions is unknown. In the near future astronauts and civilians potentially harboring undetectable micro-cancers may undertake long-term stays in space, which poses a substantial problem.

We previously reported that, in studies using our simulated microgravity-irradiation system^84,85^, simultaneous exposure of human fibroblasts to simulated microgravity and radiation had greater effects than radiation exposure alone on the expression of cell cycle-related genes associated with genomic instability^86^, and on the frequency of chromosomal aberrations^87^. It has also been reported that in a murine hindlimb suspension model simulating microgravity, hindlimb suspension significantly increased spindle cancer tumor growth and caused splenic atrophy in wild-type mice^88^. In that same study, tumor growth in severe combined immunodeficient (so-called “SCID”) mice did not differ significantly from tumor growth in wild-type controls. We also recently reported that hindlimb suspension significantly increased osteosarcoma tumor growth and metastasis to the lung, and caused greater thymic atrophy compared with mice in constant orthostatic suspension or unmanipulated mice under standard housing conditions^89^. Notably, temporary loading prevented these adverse effects^89^. Future studies should investigate irradiation-induced carcinogenesis in the hindlimb suspension model and other mouse models.

Although hindlimb suspension has been utilized in the study of adverse consequences of spaceflight, it is not a perfect model of microgravity. In addition, the mechanisms of systemic responses in the hindlimb suspension model remain unclear. Therefore, it is necessary to verify the results of hindlimb suspension studies in space-based experiments. The new mouse habitat cage units developed by JAXA enable mice to be exposed to simulated microgravity and partial gravity conditions^22^. If this device can be used in deep space, as the Deep Space Gateway habitat can, the resulting experiments will be of vital importance in clarifying the cancer risks associated with changes in gravity, in addition to reduced immunity and radiation-induced genome instability. We hope that such experiments will increase the accuracy of cancer risk assessments related to space travel.

## Probiotic and prebiotic intake as potential immune countermeasures

Crucian et al.^11,90^ and Makedonas et al.^11,90^ reviewed a range of potential immune countermeasures including exercise, stress management, medications, and nutrition/supplementation. Below, we specifically focus on probiotic and prebiotic intake as possible countermeasures. Probiotics are currently defined as “live strains of strictly selected microorganisms which, when administered in adequate amounts, confer a health benefit on the host”^91^. The effects of various kinds of probiotic supplementation on the health status of athletes have been reported^92,93^. An ongoing study by the JAXA is investigating the effects of probiotic (*Lactobacillus casei* Shirota) supplementation on intestinal microbiota and immune function in astronauts. The results of those studies will yield information on possible countermeasures.

The definition of a prebiotic was updated as a substrate that is selectively utilized by host microorganisms conferring a health benefit^94^. Fructooligosaccharides (FOS) are considered prebiotics. FOS promote the *bifidobacteria* growth^95^. In addition, FOS can indirectly and directly influence immune function through increased short chain fatty acid (SCFA) production, which modifies the interleukin production and natural killer cell activity^96^, and modification of the immune system via the gut-associated lymphoid tissue^97^. In fact, another ongoing study by JAXA is examining the effects of prebiotic (FOS) supplementation on host immunity using human and mouse specimen^44^. The results will give us more insights into countermeasure strategy. Furthermore, even synbiotics, which is a combination of synergistically acting probiotics and prebiotics introduced by Gibson and Roberfroid in 1995^98^, would be a candidate for effective countermeasures for future human exploration missions. In a prospective, randomized, double blind, placebo-controlled study on Earth, it was reported that intake of synbiotic formulations containing 3 to 5 strains of *Lactobacillus plantarum*, *Lactobacillus rhamnosus*, and *Bifidobacterium lactis*, lactoferrin and prebiotics (FOS or galactooligosaccharides) reduced the incidence and severity of URTIs during the cold season^99^. Further studies using ground analogs, for example international isolation projects and Antarctic missions, would enable to validate the countermeasure candidates.

## Data Availability

Data available on request from the authors.
